# Ulinastatin treatment for acute respiratory distress syndrome in China: a meta-analysis of randomized controlled trials

**DOI:** 10.1186/s12890-019-0968-6

**Published:** 2019-11-04

**Authors:** Xiangyun Zhang, Zhaozhong Zhu, Weijie Jiao, Wei Liu, Fang Liu, Xi Zhu

**Affiliations:** 1 Department of Pharmacy, Peking University Third Hospital, 49 North Garden Road, Beijing, 100191 China; 2 Department of Pharmacy, Henan Province Hospital of Traditional Chinese Medicine, Zhengzhou, Henan Province China; 3 Department of Epidemiology and Environmental Health, Harvard T.H. Chan School of Public Health, Boston, MA USA; 4 Department of Critical Care Medicine, Peking University Third Hospital, 49 North Garden Road, Beijing, 100191 China

**Keywords:** Ulinastatin, ARDS, RCTs, Efficacy, Meta-analysis

## Abstract

**Background:**

Epidemiologic studies have shown inconsistent conclusions about the effect of ulinastain treatment for acute respiratory distress syndrome (ARDS). It is necessary to perform a meta-analysis of ulinastatin’s randomized controlled trials (RCTS) to evaluate its efficacy for treating ARDS.

**Methods:**

We searched the published RCTs of ulinastatin treatment for ARDS from nine databases (the latest search on April 30th, 2017). Two authors independently screened citations and extracted data. The meta-analysis was performed using Rev. Man 5.3 software.

**Results:**

A total of 33 RCTs involving 2344 patients satisfied the selection criteria and were included in meta-analysis. The meta-analysis showed that, compared to conventional therapy, ulinastatin has a significant benefit for ARDS patients by reducing mortality (RR = 0.51, 95% CI:0.43~0.61) and ventilator associated pneumonia rate (RR = 0.50, 95% CI: 0.36~0.69), and shortening duration of mechanical ventilation (SMD = -1.29, 95% CI: -1.76~-0.83), length of intensive care unit stay (SMD = -1.38, 95% CI: -1.95~-0.80), and hospital stay (SMD = -1.70, 95% CI:-2.63~−0.77). Meanwhile, ulinastatin significantly increased the patients’ oxygenation index (SMD = 2.04, 95% CI: 1.62~2.46) and decreased respiratory rate (SMD = -1.08, 95% CI: -1.29~-0.88) and serum inflammatory factors (tumor necrosis factor-α: SMD = -3.06, 95% CI:-4.34~-1.78; interleukin-1β: SMD = -3.49, 95% CI: -4.64~-2.34; interleukin-6: SMD = -2.39, 95% CI: -3.34~-1.45; interleukin-8: SMD = -2.43, 95% CI: -3.86~-1.00).

**Conclusions:**

Ulinastatin seemly showed a beneficial effect for ARDS patients treatment and larger sample sized RCTs are needed to confirm our findings.

## Background

Acute respiratory distress syndrome (ARDS) is a multifactorial lung injury that continues to be associated with high levels of morbidity and mortality [1]. Although the risk of ARDS was significantly reduced over the past 20 years, its incidence is still as high as 20%~ 30% [2]. Low tidal volume ventilation, timely resuscitation and antimicrobial administration, restrictive transfusion practices, and primary prevention of aspiration and nosocomial pneumonia have likely contributed to this reduction [3]. Drug research of ARDS has been involved in all the currently recognized stages of disease pathogenesis, which included anti-inflammatory therapy of glucocorticoid, selective expansion of pulmonary vascular therapy of nitric oxide and prostacyclin, and alternative treatment of exogenous surfactant. However, no drugs are utilized in clinical practice for ARDS due to their contradictory findings across studies. Due to the lack of capable drugs for ARDS treatment, the mortality remains high, up to 45% [4]. Neutrophil and neutrophil elastase are important components of the inflammatory response that characterizes ARDS [5–7]. Current notion holds that activated neutrophil releases a large number of neutrophil elastase when it serves as a powerful host defense [5]. Neutrophil elastase is able to escape from regulation by multiple protease inhibitors at inflammatory sites. Excessive neutrophil elastase, beyond levels controlled by endogenous proteinase inhibitors, disturbs the function of the lung permeability barrier and induces the release of pro-inflammatory cytokines. Then, these actions will cause a series of symptoms that are typical in the pathophysiology of ARDS [5, 6, 8, 9]. Further supporting this proposed pathogenesis, animal model studies have shown that supplements of proteinase inhibitors reduce symptoms of acute lung injury [10]. Currently, ulinastatin is a glycoprotein that acts as a protease inhibitor and has been used to treat ARDS [11, 12], pancreatitis [13], multi-organ failure [14] and sepsis [15] in Asia for several years. A summary of the potential mechanisms accounting for the effects of ulinatistin in ARDS is shown in Table 1. For example, ulinatistin can inhibit the serine proteases (such as trypsin and α-chymotrypsin) and different enzymes (such as granulocyte elastase and hyaluronidase) as well as stabilizing lysosomal membranes to prevent the release of lysosomal enzymes [20–22]. Moreover, ulinastatin possesses anti-inflammatory properties by reducing the elevation of tumor necrosis factor-α (TNF-α), interleukin-6 (IL-6), and interleukin-8 (IL-8), which are produced by neutrophil elastase [17–19]. Currently, several animal studies [23, 24], clinical trials [19, 25] and systematic reviews [11, 12] have confirmed its beneficial effect in lung protection. However, whether ulinastatin can be recommended as a standard medication for ARDS remains uncertain, since its clinical benefit has not been fully understood [26]. Therefore, a meta-analysis to quantitatively evaluate the efficacy of ulinastatin for ARDS is essential.

**Table 1:** Potential mechanisms for the effect of ulinatistin on ARDS

ARDS risk factors	Potential mechanisms for the effect of ulinatistin on ARDS
Acute pancreatitis	Deactivation of the chain reaction of pancreatic enzymes, such as trypsin, α-chymotrypsin, lipase, amylase, elastase and carboxypeptidase [13]
Multiple organ dysfunction syndrome	Suppression of the activation of polymorphonuclear leukocytes (e.g. neutrophils), macrophages and platelets [14, 16]
Sepsis	Inhibition of various serine proteases, such as trypsin, thrombin, chymotrypsin, kallikrein, plasmin, elastase and cathepsin [15]
Systemic inflammation	Inhibition of polymorphonuclear leukocytes (e.g. neutrophils) and pro-inflammatory cytokines including interleukins (e.g. IL-1, IL-6 and IL-8) [17–19]

## Methods

### Search strategy

We searched all published randomized controlled trials (RCTs) assessing the efficacy of ulinastatin for ARDS from 9 databases: Pubmed, Ovid, the Cochrane Library, ClinicalTrials.gov, Elsevier, Web of Science, Wanfang database, China Knowledge Resource Integrated database, and VIP database. We used the following search terms: “ulinastatin” and “acute respiratory distress syndrome” or “ARDS”. The search deadline was April 30, 2017. No other restrictions were placed on the search criteria. All potentially relevant papers based on titles and abstracts were retrieved for full text screening. We also collected relevant articles by checking the references of the retrieved papers.

### Study selection

Patients, 18 years of age or older, diagnosed with ARDS were eligible for inclusion. ARDS was defined as acute onset, pulmonary artery wedge pressure ≤ 18 mmHg and absence of clinical evidence of left atrial hypertension or bilateral infiltrates on chest radiography and oxygenation index (PaO_2_/FiO_2_ ≤ 200) [27]. There were no limitations on dose or duration of ulinastatin. The intervention comparisons were made between ulinastatin plus conventional treatment and conventional treatment. The primary efficacy outcomes were mortality, rate of ventilator associated pneumonia (VAP), duration of mechanical ventilation, length of intensive care unit (ICU) and hospital stay. Secondary efficacy outcomes were PaO_2_/FiO_2_, respiratory rate, serum inflammatory factors (TNF-a, IL-1β, IL-6, IL-8).

### Data extraction and quality assessment

Both the study selection (XYZ and WJ) and data extraction processes (XYZ and WL) were performed by two authors independently. Disagreements were resolved through consensus or arbitration by a third author (XZ or FL). Data for basic characteristics of included trials extracted from full-text articles included the following terms: first author, year of publication, mean age or range, the number of patients, intervention information, Jadad score and duration of intervention. We obtained mean ± standard deviation values for continuous variables in the original manuscripts for the meta-analysis. Trials in which specific endpoints were not reported were excluded from the pooled analyses of the specific endpoints that were reported. Study quality was assessed by the Jadad scale, which assesses adequacy of randomization, blinding and attrition. The Jadad scale ranges from 0 to 5 points, with a low-quality study receiving a score of 2 or less and a high-quality study having a score of at least 3 [28].

### Statistical analysis

Data analyses were performed in Rev. Man 5.3 software. The results were expressed as standard mean difference (SMD), weighted mean difference (WMD), relative risk (RR) with 95% confidence interval (CI), I^2^ value, and Egger test’s *P* value. I^2^ value serves as a marker of inter-trial heterogeneity [29]. If I^2^ < 50%, the fixed-effect model (Mantel-Haenszel) was employed without considering inter-trial heterogeneity. Otherwise, sensitivity analyses were used to identify the sources of inter-trial heterogeneity. In sensitivity analyses, we serially left one study out and analyzed heterogeneity on the basis of masking within the trial in order to judge the stability of effective values. If effective values were stable in sensitivity analyses, the random-effect model (Inverse Variance) was used. If effective values were unstable in sensitivity analyses, we tended to give up performing a meta-analysis and just made a descriptive analysis. Finally, publication bias was formally assessed by using funnel plot and Egger’s regression analysis (with *P* < 0.05 defined as having publication bias [30]).

## Results

### Study characteristics

**Fig. 1:**
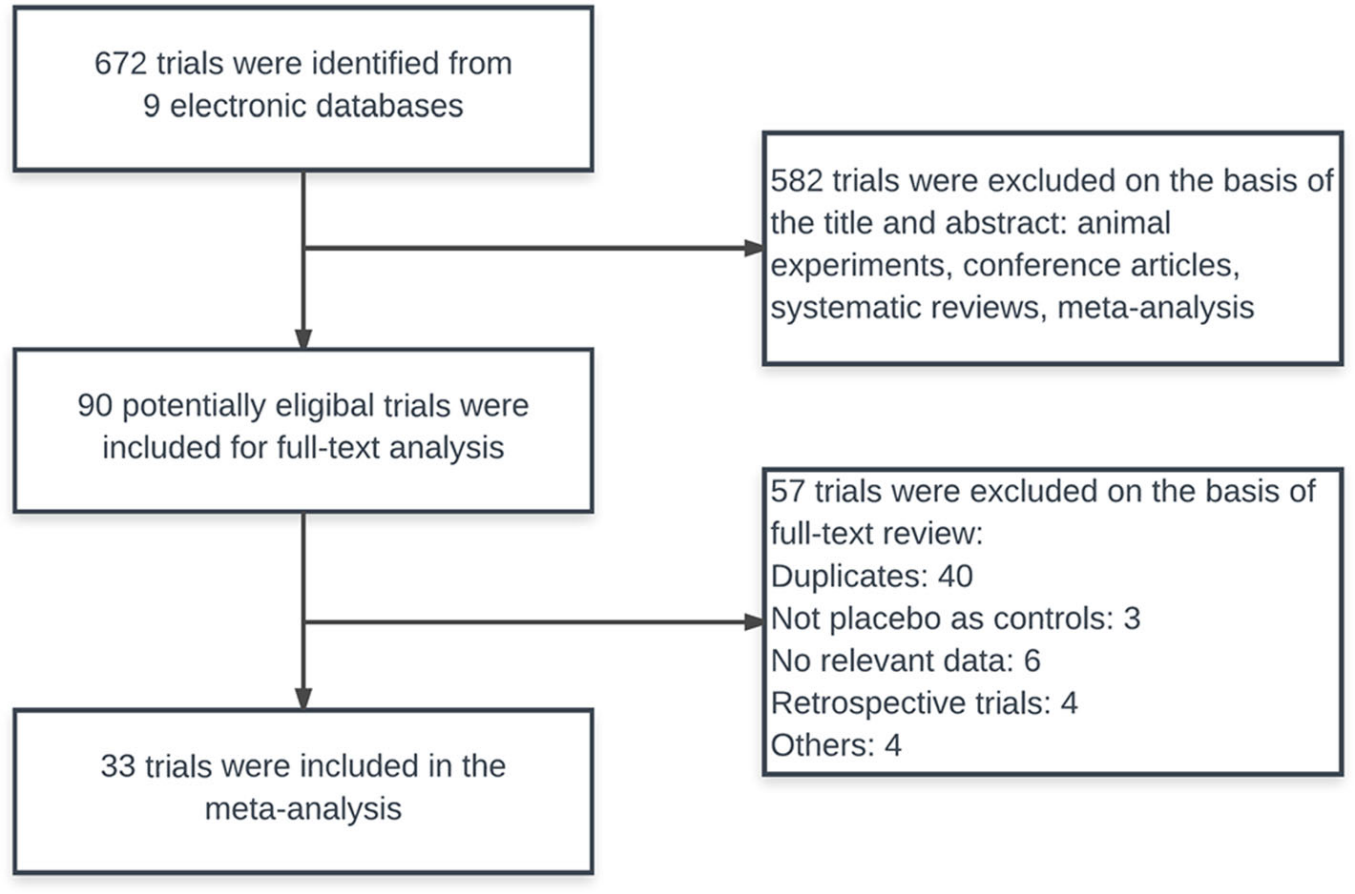
Literature search results for publications related to randomized controlled trials of ulinastatin treatment for acute respiratory distress syndrome

A total of 672 potentially relevant RCTs were identified and screened, using the process shown in Fig. 1. We retrieved 90 RCTs for detailed evaluation, out of which 33 RCTs involving 2344 patients satisfied the selection criteria [31–63]. The included trials were published between 2003 and 2017. The median number of patients was 71 (range 36–160), with three trials [47, 48, 61] having more than 100 patients. Treatment duration ranged from 3 to 30 days. The conventional therapy included mechanical ventilation, anti-infective therapy, nutritional support, treatment of primary diseases, etc. Although all the trials announced the randomization, 7 RCTs adequately described randomization procedures [31, 33, 38, 42, 43, 45, 49, 61], and 1 study [60] explicitly mentioned the method of allocation concealment using opaque envelope. Table 2 displays the quality and characteristics of these studies.

**Table 2:** Characteristics and quality of all included studies

Author	Year	Intervention^a^	Age/year (Range or SD)	Total (T/C)	Duration/d	Jadad Score
Zhu.GY [58]	2003	ulinastatin, 20w U, ivgtt, bid	28.0~61.0	61 (31/30)	5	2
Xue.HX [50]	2008	ulinastatin, 60w U, ivgtt, q6h	20.0~81.0	60 (30/30)	7	2
Ou.SQ [53]	2008	ulinastatin, 20~30w U, ivgtt, bid	45.0~80.0	36 (18/18)	5~7^b^	2
Hu.MH [59]	2009	ulinastatin, 30w U, ivgtt, q8h	41.2 ± 18.4	54 (29/25)	7	2
Zhang.YL [35]	2009	ulinastatin, 20w U, iv, bid	61.9 ± 17.5	61 (31/30)	7	2
Zhang.CQ [39]	2010	ulinastatin, 50w U, ivgtt, bid	55.7 ± 15.6	60 (30/30)	7	2
Tan.C [52]	2010	ulinastatin, 20w U, iv, qd	39.2 ± 6.9	50 (25/25)	7	2
Zhang.CG [34]	2011	ulinastatin, 20w U, ivgtt, q12h	18.0~65.0	82 (42/40)	7	2
Zhou.MH [36]	2011	ulinastatin, 60w U, ivgtt, qid	40.2 ± 5.3	40 (20/20)	5	2
Liu.JX [41]	2012	ulinastatin, 30w U, ivgtt, bid	57.0 ± 16.7	78 (40/38)	6	2
He.B [47]	2012	ulinastatin, 20w U, iv, q8h	53.9 ± 11.8	104 (52/52)	10	2
Gu.JP [48]	2012	ulinastatin, 30w U, cip(1 h), bid	61.5 ± 31.6	160 (76/84)	7	2
Ye.QD [57]	2012	ulinastatin, 20w U, ivgtt, q8h	51.0 ± 12.8	84 (42/42)	14	2
Hu.Y [63]	2014	ulinastatin, 20w U, ivgtt, bid	37.9 ± 2.4	60 (30/30)	7	2
Zeng.BL [31]	2014	ulinastatin, 20w U, ivgtt, tid	18.0~51.0	60 (30/30)	7	3
Tian.ZT [32]	2014	ulinastatin, 20w U, ivgtt, tid	53.9 ± 8.2	90 (45/45)	7	3
Cao.YY [33]	2014	ulinastatin,4w U, ivgtt, tid	17.0~72.0	68 (34/34)	7	3
Ji.MX [38]	2014	ulinastatin, 10w U, ivgtt, tid	24.0~79.0	80 (40/40)	7	3
Ding.HH [44]	2014	ulinastatin, 20w U, ivgtt, bid	24.0~85.0	68 (36/32)	7	2
Huang.HT [51]	2014	ulinastatin, 20w U, cip, bid	42.6 ± 8.9	62 (29/33)	7	2
Lin.B [62]	2015	ulinastatin, 10w U, iv, bid	16.0~68.0	44 (22/22)	7~10^b^	2
Liu.YX [61]	2015	ulinastatin, 10w U, iv, bid	20.0~67.0	100 (50/50)	7	3
Duan.PL [60]	2015	ulinastatin, 20w U, ivgtt, tid	19.0~65.0	70 (35/35)	7	3
Huang.ZX [40]	2015	ulinastatin, 30w U, ivgtt, q6h	52.3 ± 7.1	58 (30/28)	3	2
Yan.ZH [43]	2015	ulinastatin, 20w U, ivgtt, bid	20.0~63.0	73 (37/36)	7	3
Wei.M [45]	2015	ulinastatin, 20w U, ivgtt, q8h	47.1 ± 7.1	94 (48/46)	7	3
He.C [55]	2015	ulinastatin, 3w U, ivgtt, qid	53.1 ± 7.0	48 (30/18)	3	2
Miu.SX [42]	2016	ulinastatin, 200w U, ivgtt, bid	28.0~75.0	62 (31/31)	7	3
Huang.QS [46]	2016	ulinastatin, 60w U, ivgtt, qd	50.5 ± 6.0	76 (38/38)	7	2
Mo.ZM [49]	2016	ulinastatin, 20w U, iv, tid	29.0~72.0	81 (41/40)	10	3
Wu.YQ [54]	2016	ulinastatin,10w U, iv, q8h	26.0~82.0	80 (40/40)	5	2
Ye.YY [56]	2016	ulinastatin, 60w U, ivgtt, q8h	18.0~71.0	80 (40/40)	12	2
Wang.ZH [37]	2017	ulinastatin, 20w U, ivgtt, q8h	28.0~71.0	60 (30/30)	7	2

*Abbreviations*: *ivgtt* Intravenously guttae, *iv* Intravenous, *cip* Continuous intravenous pumping, *1w U* 10,000 units, *T* The number of test group, *C* The number of control group, ^a^ The conventional treatment was same in both the experimental and control groups, including mechanical ventilation, anti-infective therapy, organ support, and treatment of primary diseases, etc. The experimental group was treated with ulinastatin on the basis of conventional treatment; ^b^ The duration of ulinastatin treatment applicated varies with each patient’s condition

### Primary efficacy outcomes

**Fig. 2:**
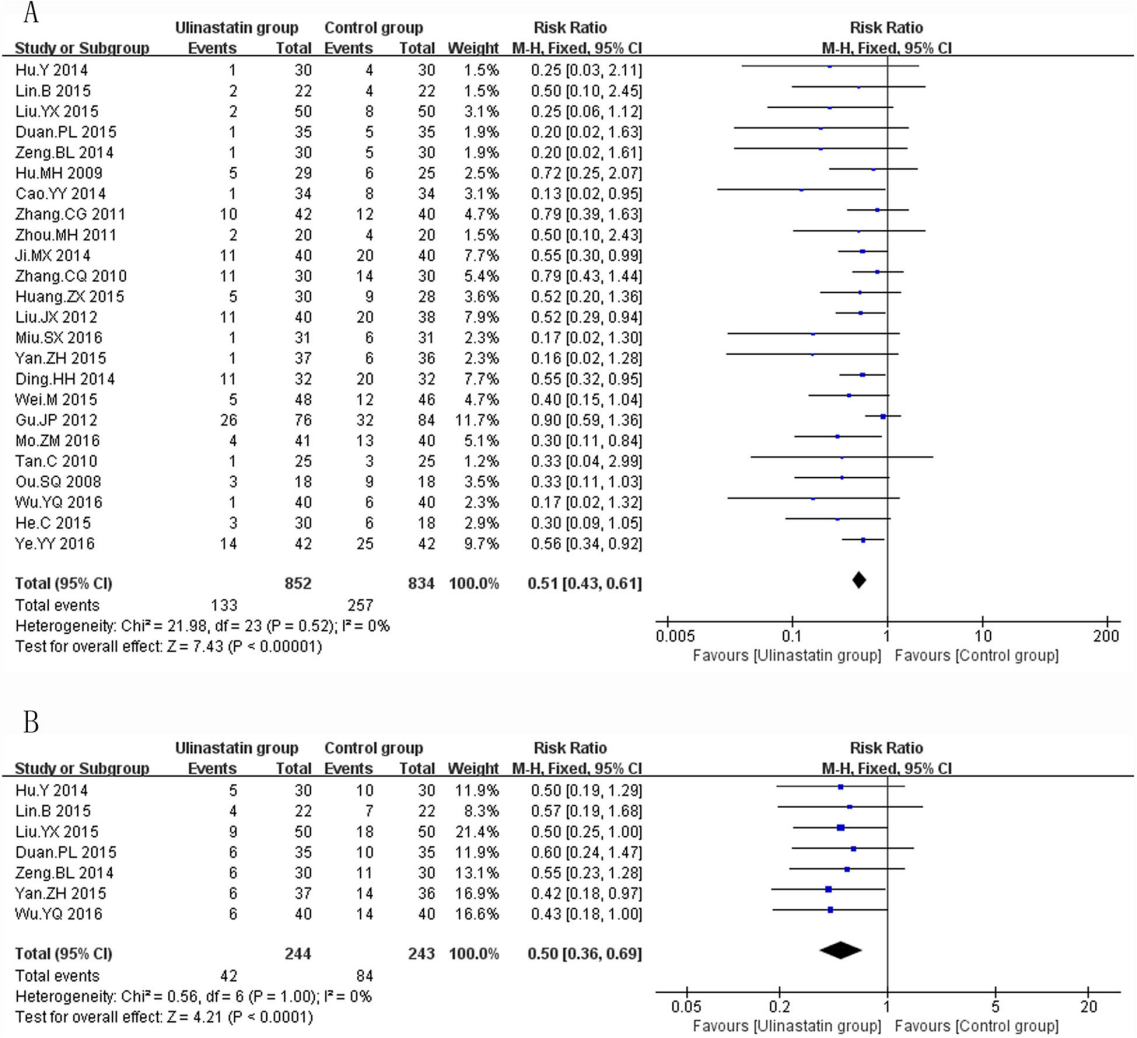
The meta-analysis results of patients’ mortality (**a**) and ventilator associated pneumonia rate (**b**) after treatment

**Fig. 3:**
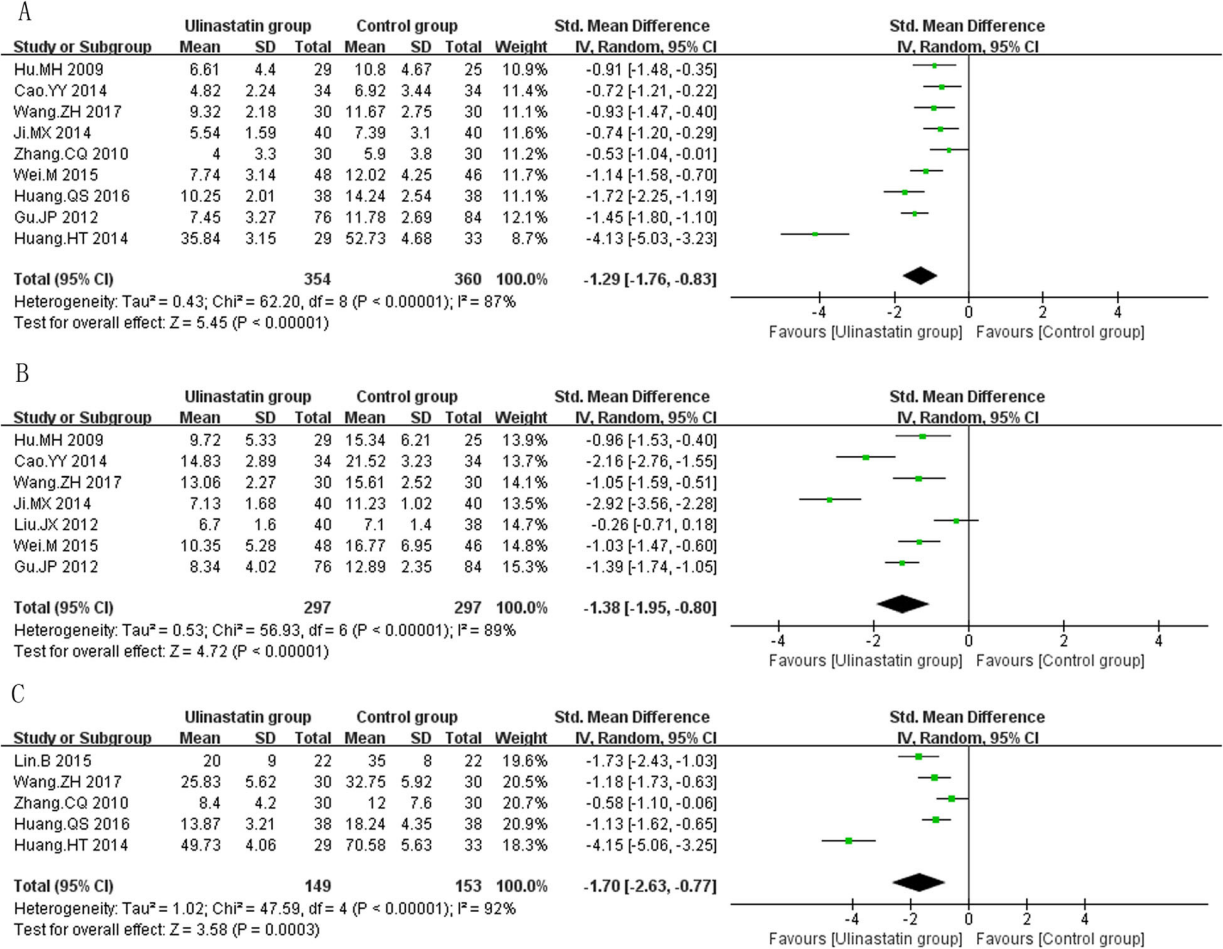
The meta-analysis results of patients’ duration of mechanical ventilation (**a**), intensive care unit stay (**b**) and hospital stay (**c**) after treatment

The primary efficacy outcomes on which we focused were directly associated with clinical benefit, including mortality and VAP rate, duration of mechanical ventilation, and length of stay (ICU stay, hospital stay). A total of 24 RCTs [31, 33, 34, 36, 38–45, 48, 49, 52–56, 59–63] (1686 patients) reporting patients’ mortality, the results of meta-analysis confirmed that ulinastatin significantly decreased mortality (RR = 0.51, 95% CI: 0.43~0.61, *P* < 0.0001, I^2^ = 0%, *P*_egger_ < 0.001, Fig. 2a). Similarly, in a total of 7 RCTs [31, 43, 54, 60–63] (487 patients) reporting patients’ VAP rate, the results of meta-analysis found that ulinastatin significantly decreased patients’ VAP rate (RR = 0.50, 95% CI: 0.36~0.69, *P* < 0.0001, I^2^ = 0%, *P*_egger_ = 0.873, Fig. 2b). Moreover, ulinastatin also significantly shortened duration of mechanical ventilation (SMD = -1.29, 95% CI: -1.76~-0.83, *P* < 0.0001, I^2^ = 87%, *P*_egger_ = 0.221, 9 RCTs including 714 patients [33, 37–39, 45, 46, 48, 51, 59] Fig. 3a), ICU stay (SMD = -1.38, 95% CI: -1.95~-0.80, *P* < 0.0001, I^2^ = 89%, *P*_egger_ = 0.339, 7 RCTs including 594 patients [35, 38, 42, 43, 46, 50, 53], Fig. 3b), and hospital stay (SMD = -1.70, 95% CI: -2.63~-0.77, *P* < 0.0001, I^2^ = 92%, *P*_egger_ = 0.029, 5 RCTs including 302 patients [37, 39, 46, 51, 62], Fig. 3c). The overall results were similar after sequentially excluding each individual study. The test for heterogeneity was not significant for mortality and VAP rate and fixed-effects model was used. However, there was significant inter-trial heterogeneity in duration of mechanical ventilation, ICU stay and hospital stay and random effect model was used. Egger’s regression analysis found publication bias existed in mortality and hospital stay.

### Secondary efficacy outcomes

Secondary efficacy outcomes on which we focused were indirectly associated with clinical benefit, including PaO_2_/FiO_2_, respiratory rate, and serum inflammatory factors (TNF-a, IL-1β, IL-6, IL-8). A total of 26 RCTs [31, 32, 34–38, 40–46, 48, 50, 52, 53, 55–57, 59–63] including 1824 patients reported PaO_2_/FiO_2_. Compared with conventional therapy, ulinastatin significantly increased patients’ PaO_2_/FiO_2_ (SMD = 2.04, 95% CI: 1.62~2.46, *P* < 0.00001, I^2^ = 93%, *P*_egger_ < 0.001), which was confirmed by the results of meta-analysis (Table 3). Moreover, the findings of meta-analysis on patients’ secondary efficacy outcomes after ulinastatin treatment (Table 3) suggested that ulinastatin significantly decreased patients’ respiratory rate (SMD = -1.08, 95% CI: -1.29~-0.88, *P* < 0.0001, I^2^ = 60%, *P*_egger_ = 0.001, 15 RCTs including 1117 patients [31, 34–36, 43, 47–50, 52–54, 60, 61, 63]) and serum inflammatory factors (TNF-α: SMD = -3.06, 95% CI: -4.34~-1.78, *P* < 0.0001, I^2^ = 97%, *P*_egger_ < 0.001, 8 RCTs including 600 patients [33, 43–46, 56–58]; IL-1β: SMD = -3.49, 95% CI: -4.64~-2.34, *P* < 0.0001, I^2^ = 78%, 2 RCTs including 137 patients [46, 58]; IL-6: SMD = -2.39, 95% CI: -3.34~-1.45, *P* < 0.0001, I^2^ = 94%, *P*_egger_ = 0.002, 7 RCTs including 523 patients [33, 39, 45, 46, 56–58]; IL-8: SMD = -2.43, 95% CI: -3.86~-1.00, *P* < 0.0001, I^2^ = 95%, *P*_egger_ = 0.015, 4 RCTs including 286 patients [43, 44, 57, 58]). Though significant heterogeneity existed, the overall results of all the outcomes were similar after sequentially excluding each individual study. Egger’s regression analysis found publication bias existed in these outcomes.

**Table 3:** The meta-analysis results of patients’ secondary efficacy outcomes after treatment

Outcome indexs	No.Trials (Patients)	SMD (95% CI)	I^2^ (%)	P_Egger_
Oxygenation index (PaO_2_/FiO_2_)	26 (1824)	2.04 [1.62, 2.46]	93	< 0.001
Respiratory rate	15 (1117)	-1.08 [−1.29, − 0.88]	60	0.001
Serum inflammatory factor (TNF-α)	8 (600)	-3.06 [−4.34, −1.78]	97	< 0.001
Serum inflammatory factor (IL-1β)	2 (137)	-3.49 [−4.64, − 2.34]	78	–
Serum inflammatory factor (IL-6)	7 (523)	-2.39 [−3.34, −1.45]	94	0.002
Serum inflammatory factor (IL-8)	4 (286)	-2.43 [−3.86, −1.00]	95	–

*Abbreviations*: *SMD* Standardized mean difference

## Discussion

ARDS is a syndrome with acute lung and systemic inflammation, which are because of activation and accumulation of neutrophils and cytokines [7, 64]. ARDS remains a major public health problem that incurs high health care costs and causes major mortality in ICU despite some improvements in conventional therapeutic approach and managements in the past decades, including mechanical ventilation, systemic steroid, and nitric oxide [65]. Ulinastatin, known as a protease inhibitor, is found in the urine, plasma and all organs [66] and has been used to treat acute pancreatitis [67], acute circulatory failure [68], and severe sepsis [69, 70]. However, it remains uncertain whether ulinastatin can be recommended as a standard medication for and ARDS. In animal models, ulinastatin attenuates the pathophysiological process of acute lung injury induced by lipopolysaccharide, scald injury, phosgene and seawater, among other injuries [23, 24, 71–73]. These benefits are mainly associated with inhibiting the activation of neutrophils, blocking nuclear factor-κB pathway, which plays a critical role in the regulation of pro-inflammatory (e.g. TNF-α, IL-1β, IL-6), down-regulate chemokines (e.g. IL-8, macrophage inflammatory protein-2), and increases neutrophils apoptosis [5, 23, 24, 67, 68, 71–73]. In theory, ulinastatin could be a new option in ARDS treatment. Several clinical trials and systematic reviews [11, 12] have also confirmed the lung protection of ulinastatin. We performed this meta-analysis to present a comprehensive evaluation to date of ulinastatin in patients with ARDS.

The primary efficacy outcomes were directly associated with clinical benefit, including mortality and VAP rate, duration of mechanical ventilation, and length of stay (ICU stay, hospital stay). Over the past decades, a significant decline has been found in mortality of ARDS, but still as high as 45% [4]. The majority of deaths are attributable to sepsis or multiple organ dysfunction rather than primary respiratory causes, but in some cases death is directly related to lung injury [64]. Ulinastatin has an exact lung protection pharmacological mechanism. In our study, compared to conventional therapy, the results clearly showed that ulinastatin could reduce mortality by 49%, which provided convincing evidence that the pharmacological effect of ulinastatin could be translated into a clinical benefit. Additionally, the results provided more evidence to prompt ulinastatin to become a new hope for ARDS treatment. Ulinastatin significantly reduced patients’ VAP rate by 50% and shorten duration of mechanical ventilation (WMD = -4.60 days, 95% CI: -6.83 ~-2.37), which contributed to a reduced risk of death from another perspective. Ulinastatin also shorten more patients’ hospital stay (WMD = -10.09 days, 95% CI: -17.24 ~-2.94) than ICU stay (WMD = -4.18 days, 95% CI: -5.98 ~-2.38), so it might be able to drastically reduce the cost of medical treatment for patients and government.

Secondary efficacy outcomes were indirectly associated with clinical benefit, including PaO_2_/FiO_2_, respiratory rate, and serum inflammatory factors (TNF-α, IL-1β, IL-6, IL-8). Our meta-analysis result found ulinastatin could increase patients’ PaO_2_/FiO_2,_ and the improvement of PaO_2_/FiO_2_ has been suggested to be positively related to mortality [65], which was consistent with our findings. Moreover, our study showed ulinastatin could decrease respiratory rate and serum inflammatory factors (TNF-α, IL-1β, IL-6, IL-8), which was consistent with animal models’ results [24, 73, 74]. Although, our study supported ulinastatin to be an effective treatment for ARDS, we found an older study [75] indicates intra-alveolar ulinastatin cannot inhibit polymorphonuclear elastase activity in the lung in postsurgical patients with ARDS. The statistical significance of this conclusion is questionable as the sample size is too small (only 8 patients). Compared to this study, our study increased the sample size to include 2344 patients and took into account multiple clinically relevant outcomes, so our findings were robust and more reliable.

Although, our study suggested that ulinastatin was relatively effective for the treatment of ARDS and provided a justification for large, well-designed, RCTs to examine the effects of ulinastatin in ARDS, by limiting the study population to patients with ARDS, and increasing the sample size, and expanding the research outcomes, but there were still several limitations. First, publication bias existed in mortality and secondary efficacy outcomes, which probably stemmed from small-study effects [76], all of the trials published in Chinese and the exclusion of trials published as abstracts and conference articles. Second, significant heterogeneity was shown for all the continuous outcomes (ie, duration of mechanical ventilation, ICU stay, hospital stay). Mostly, potential sources of heterogeneity have been identified by sensitivity analyses and random effect model, but some residual heterogeneity still existed in this meta-analysis, which might have originated from the included studies’ low quality (most studies’ Jadad score of only 2), small sample size (range 36–160), or variations in conventional interventions. Therefore, more large-scale multicenter, well designed, RCTs are needed to verify ulinastatin’s efficacy. Lastly, no information on safety was provided by the trials included in our meta-analysis, which is especially needed for future studies.

## Conclusions

The findings of this meta-analysis seemly support ulinastatin to be an effective treatment for ARDS. This drug might reduce mortality, ventilator associated pneumonia rate, shortening duration of mechanical ventilation, length of ICU stay and hospital stay in ARDS patients, which needs large, well-designed, RCTs to confirm.

## Data Availability

The datasets analyzed during the current study are available from the corresponding author on reasonable request.

## Abbreviations

ARDS: Acute respiratory distress syndrome
CI: Confidence interval
ICU: Intensive care unit
IL-6: Interleukin-6
IL-8: Interleukin-8
PaO_2_/FiO_2_: Ratio of arterial oxygen partial pressure to fractional inspired oxygen
RCTs: Randomized controlled trials
RR: Relative risk
SMD: Standard mean difference
TNF-α: Tumor necrosis factor-α
VAP: Ventilator associated pneumonia
WMD: Weighted mean difference
